# Treatment of IgG4-related disease-associated hypertrophic pachymeningitis with intrathecal rituximab: a case report

**DOI:** 10.3389/fneur.2023.1189778

**Published:** 2023-05-24

**Authors:** Denis T. Balaban, Spencer K. Hutto, Bruno P. Panzarini, Aileen O'Shea, Aditi Varma, Pamela S. Jones, Bart K. Chwalisz, John H. Stone, Nagagopal Venna

**Affiliations:** ^1^Division of Neuroimmunology and Neuroinfectious Disease, Department of Neurology, Massachusetts General Hospital, Harvard Medical School, Boston, MA, United States; ^2^Division of Hospital Neurology, Department of Neurology, Emory University School of Medicine, Atlanta, GA, United States; ^3^Department of Radiology, Massachusetts General Hospital, Harvard Medical School, Boston, MA, United States; ^4^Department of Neurosurgery, Massachusetts General Hospital, Harvard Medical School, Boston, MA, United States; ^5^Neuro-Ophthalmology, Massachusetts Eye and Ear Infirmary, Boston, MA, United States; ^6^Department of Rheumatology, Massachusetts General Hospital, Harvard Medical School, Boston, MA, United States

**Keywords:** IgG4-related disease, pachymeningitis, hydrocephalus, intrathecal rituximab, CSF IgG4, optic neuropathy

## Abstract

IgG4-related disease-associated hypertrophic pachymeningitis (IgG4RD-HP) is a fibroinflammatory autoimmune disorder in which diagnosis is difficult without biopsy. Guidance on management of disease refractory to glucocorticoids and intravenous rituximab is limited. We present the case of a 68-year-old woman with IgG4RD-HP who developed sensorineural hearing loss with associated bulky basilar pachymeningeal enhancement. Her cerebrospinal fluid was inflammatory and had an elevated IgG4 concentration, strongly suggestive of IgG4RD-HP. Biopsy of involved meninges was not possible due to surgical risk. Over years she developed bilateral optic neuropathies and hydrocephalus, requiring intravenous rituximab and ventriculoperitoneal shunt. Her disease was refractory to glucocorticoids. Despite maintenance intravenous rituximab, she developed slowly progressive symptoms of intracranial hypertension and hydrocephalus with persistently inflammatory spinal fluid. Switching to intrathecal rituximab therapy led to dramatic improvement in gait and headache and reduced pachymeningeal bulk and metabolic activity. In patients with IgG4RD-HP refractory to glucocorticoids and intravenous rituximab, intrathecal rituximab may be an efficacious therapy.

## 1. Introduction

IgG4-related disease-associated hypertrophic pachymeningitis (IgG4RD-HP) is a fibroinflammatory disorder in which a combination of peripheral and intrathecal inflammation may play a role (1). The first-line treatment for IgG4-RD is glucocorticoids. Intravenous rituximab is a second-line therapy but has limited ability to penetrate the blood-brain barrier. Intrathecal rituximab has been reported to lead to clinical improvement in IgG4-RD-HP (2, 3). We report a patient with IgG4RD-HP who developed hearing loss, optic neuropathy, and hydrocephalus and experienced clinicoradiographic improvement on intrathecal rituximab after failure of intravenous rituximab.

## 2. Case history

**Figure 1:**
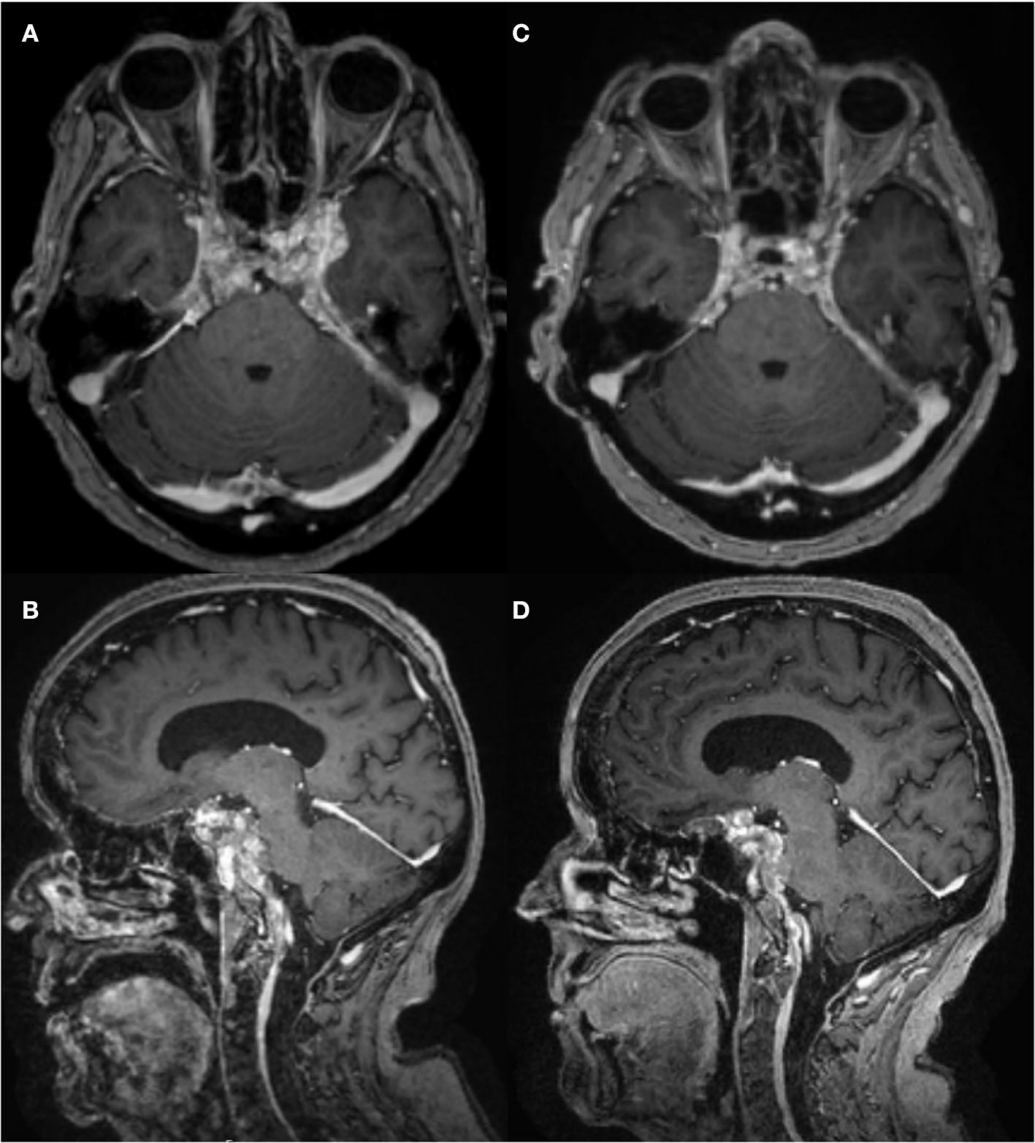
Brain MRI T1 post-contrast, pre- and post-intrathecal rituximab. **(A, B)** January 2021, before intrathecal rituximab. **(C, D)** March 2022, after two cycles of intrathecal rituximab. There was mild improvement in enhancement and pachymeningeal bulk after cycle 1, but not after cycle 2.

In March 2012, a 68-year-old woman developed left-sided sensorineural hearing loss confirmed by audiogram. Brain MRI showed dural-based enhancing nodules involving the prepontine and perimesencephalic cisterns (Figures 1A, B). The lesion encased the basilar artery and internal carotid arteries and also involved the left internal auditory canal, suprasellar cistern abutting the optic chiasm, orbital apices, and bilateral cavernous sinuses. Other than left-sided hearing loss, cranial nerves, strength, reflexes, sensation, and gait were normal. Initial serum, CSF, and systemic imaging studies excluded infectious, autoimmune, and neoplastic mimics (Table 1). She had no oral or genital ulcers. Serum IgG4 was elevated at 128 mg/dL (normal range 3.9–86.4). A CSF examination from September 2012 demonstrated 79 nucleated cells/mcL (97% lymphocytes), an elevated protein concentration of 148 mg/dL (normal 5–55 mg/dL), and a CSF glucose of 49 mg/dL (normal 50–75 mg/dL). Oral prednisone 60 mg daily and methotrexate 15 mg weekly were begun to treat inflammatory pachymeningitis suspicious for IgG4RD-HP.

**Table 1:** Diagnostic testing investigating for infectious and inflammatory mimics of IgG4-related disease.

**Serum test**	**Value**	**CSF test**
Antinuclear antibody	1:80, nucleolar pattern (normal < 1:40)	Cytology without malignant cells
C3	125 (normal 86–184 mg/dL)	VDRL negative
C4	22 (normal 16–38 mg/dL)	Cryptococcal antigen negative
Erythrocyte sedimentation rate	13 mm/h	Mycobacterial culture without growth
ANCA	Negative	
SS-A, SS-B	Negative	Imaging
Anti-Smith	2.53 (normal 0–19.99)	CT chest/abdomen/pelvis showed no lesions suggestive of sarcoidosis or malignancy.
Anti-RNP	0.46 (normal 0–19.99)	
Anti-dsDNA	Negative	
Anti-Scl70	1.13 (normal 0–19.99)	
Rheumatoid factor	< 30	
Interferon gamma release assay	Negative	
Tuberculin skin test	No induration	
Anti-mitochondrial	Negative	
Anti-tTG IgA	2.3 (normal 0–15 U/mL)	

Following her prednisone taper, in October 2014 she developed right afferent pupillary defect and worsening pinhole corrected visual acuity (20/30 OD, 20/40 OS) and was diagnosed with an optic neuropathy. January 2015 CSF showed 107 nucleated cells/mcL (98% lymphocytes), protein 181 mg/dL, glucose 56 mg/dL. The CSF IgG4 was 5.06 mg/dL and the serum IgG4 was 111.2 mg/dL. A diagnosis of IgG4RD-HP was made based on clinicoradiographic presentation and elevated CSF IgG4 based on previously published diagnostic cutoff of 2.27 mg/dL (4).

**Figure 2:**
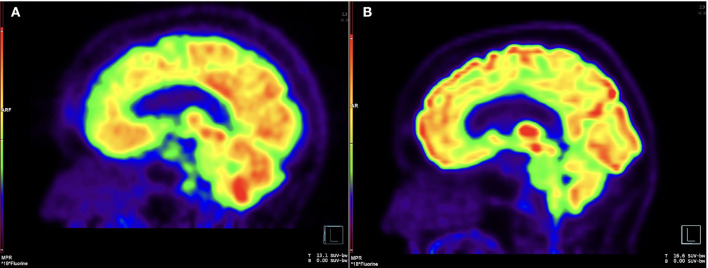
Brain FDG-PET, pre- and post-intrathecal rituximab. **(A)** Sagittal image from 18F-FDG PET before intrathecal rituximab demonstrates diffuse, patchy cerebral cortical hypometabolism and intense nodular uptake along the retroclival region, corresponding to areas of pachymeningeal disease. **(B)** Follow-up imaging after intrathecal rituximab demonstrates interval resolution of previously seen cortical hypometabolism and abnormal retroclival uptake.

Vision deteriorated to count fingers OD over months. Rituximab 1,000 mg IV every 2 weeks for the first two doses replaced methotrexate in March 2015. In July 2015 she developed obstructive hydrocephalus resulting in unsteady gait with magnetic quality and subsequent fall. Ventriculoperitoneal shunt placement in August 2015 improved her gait. During shunt placement, biopsy of brain and dura uninvolved by pachymeningitis showed non-specific reactive gliosis. She continued intravenous rituximab every 4 months. Pinhole corrected visual acuity improved to 20/60 OD with inferior altitudinal defect and 20/25 OS with temporal hemianopsia. In February 2020, she developed morning headaches worsened by supine position. Lumbar puncture June 2020 showed opening pressure 18 cm H2O, 23 nucleated cells/mcL, protein 727 mg/dL, and elevated IgG4 17.4 mg/dL with serum IgG4 74 mg/dL. X-rays showed intact shunt. Shunt was reprogrammed with symptomatic relief, but symptoms recurred in December 2020. Brain FDG-PET showed hypermetabolism in areas of pachymeningitis (Figure 2A).

The patient's gait grew more unsteady, such that she had to walk with a walker at all times. Due to her persistent headache, gait worsening, pachymeningitis, and intrathecal inflammation, a decision to escalate therapy to intrathecal ritixumab was made. She underwent shunt revision in January 2021 with addition of on/off valve to prevent CSF drainage to peritoneal cavity in preparation for intrathecal rituximab administration via the shunt.

In March 2021, 2 days prior to receiving intrathecal rituximab, visual acuity with pinhole correction was 20/30−2 OD with inferior altitudinal defect and 20/25−2 OS with temporal hemianopsia. Intrathecal rituximab was administered according to the following protocol: the shunt valve was turned off for 30 min to allow for spinal fluid to accumulate, after which 15 mL of CSF was drained, 10 mg of rituximab was administered via the shunt, and 6 mL of CSF administered to flush the tubing. After 1 h, the shunt valve was turned back on to allow for normal drainage. This protocol was repeated every 2 weeks for total of four sessions, occurring every 6 months.

**Figure 3:**
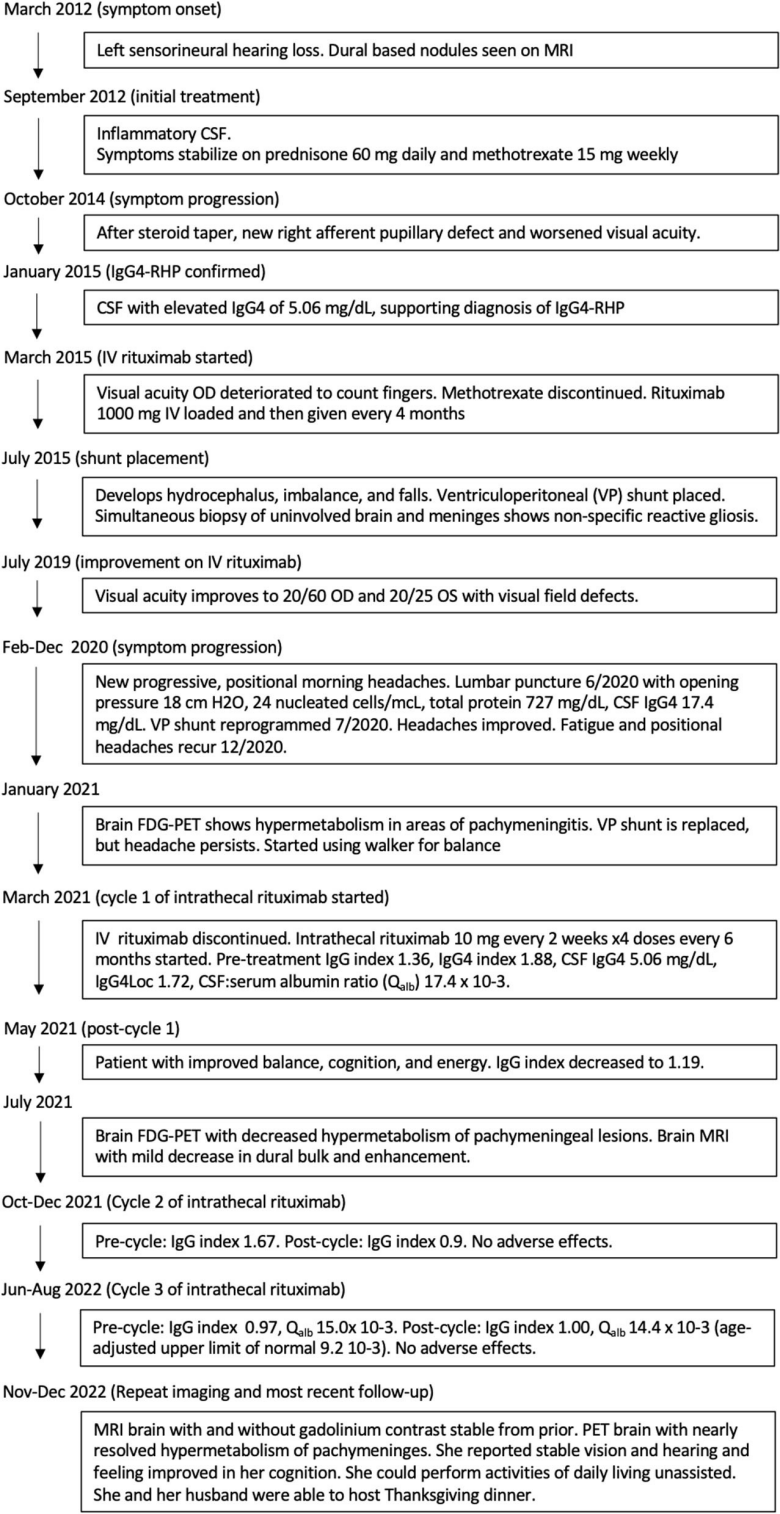
Flowchart of the patient's clinical course of treatment.

CSF drawn before the first treatment showed elevated CSF IgG index 1.36, IgG4 index 1.88, IgG4 2.7 mg/dL, IgG4Loc 1.72, and CSF:serum albumin ratio 17.4 x 10-3. She tolerated intrathecal rituximab well with facial (Q_alb_) flushing as the only side-effect. She felt remarkable improvement in her ability to think clearly, an increase in her energy level, and vastly improved steadiness of gait and turning ability. IgG index in May 2021 decreased to 1.19. Brain PET July 2021 showed mild decreased hypermetabolism of the pachymeninges. Brain MRI showed mild decrease in dural bulk and enhancement with less encasement of the basilar and internal carotid arteries. In October 2021, her visual acuity with pinhole correction improved to 20/30 +1 OD and 20/25 +1 OS with persistent visual field defects as before. IgG index at beginning of second cycle in October 2021 was 1.67, which reduced to 0.9 in December 2021. Brain MRI in March 2022 showed no decrease in pachymeningeal bulk. At January 2022 follow-up, she reported no headaches nor falls saying, “I feel like I am enjoying life the way I am supposed to.” November 2022 brain MRI was stable (Figures 1C, D). Brain PET showed nearly resolved hypermetabolism of the pachymeninges (Figure 2B). She has continued to improve, and at last follow-up in December 2022 she had a normal gait and was able to walk down a long hallway without a cane. Figure 3 provides a summary of the patient's history, testing and treatment over time.

## 3. Discussion

This case report describes the protocol used for intrathecal rituximab administration in a case of refractory IgG4RD-HP, a treatment that led to an excellent outcome and was remarkably well tolerated. IgG4RD is a multisystemic fibroinflammatory disorder. Diagnosis is the assimilation of information from the clinical examination, serological evaluation, radiologic evidence, and pathology findings (5). It is also important to exclude potential mimickers. Diagnosis of the disease involving the pachymeninges and CNS is frequently challenging, particularly if the sites of disease pose challenges to biopsy, as seen in our case. Standard treatment of IgG4-RD includes glucocorticoids (5, 6). Therapies targeting B-lymphocytes, particularly the intravenous administration of rituximab, have also demonstrated good success in a high percentage of IgG4-RD patients (7).

In IgG4RD-HP, disease is often isolated to the meninges and inflammation can be restricted to the intrathecal compartment, making measurement of CSF IgG4 markers a useful diagnostic adjunct (4, 8). Cutoffs for such markers have been suggested as alternatives to biopsy when it is contraindicated or uninformative, like in our patient (4). Our patient's values for CSF IgG4 (5.06 mg/dL >2.27 mg/dL) and IgG4Loc (1.72 >0.47) were above these cutoffs, which helped rule in IgG4RD-HP after other disorders were excluded (4, 9).

Despite initial improvement and stabilization with ventriculoperitoneal shunt and intravenous rituximab, her symptoms ultimately progressed. One possible explanation for this is the failure of intravenous rituximab to target intrathecal inflammation. Intravenous rituximab achieves 0.1–0.5% CSF concentration as compared to that of serum (3, 10). Thus, in some IgG4RD-HP patients who respond to intravenous rituximab, it is possible this small amount of penetrance may be enough to control disease. However, patients refractory to intravenous rituximab may have greater response to the higher effective dose that intrathecal rituximab provides.

Two reports in the literature describe successful treatment of IgG4RD-HP with intrathecal rituximab after failure of intravenous rituximab (2, 3). In both cases, the patients had frontal pachymeningitis, a location that affords lower surgical risk, allowing for biopsy confirmation. Both received intrathecal rituximab < 3 years after diagnosis and demonstrated marked improvement in pachymeningeal enhancement and associated parenchymal T2 hyperintensities. One of the cases also described normalization of CSF:serum albumin ratio (QQ_alb_), a measure of CSF flow rate and blood-CSF barrier dysfunction (2, 9). Neither measured CSF IgG4 markers or described adverse effects from intrathecal rituximab.

Our report differs in that the patient carried the diagnosis for 9 years before intrathecal rituximab administration, had bulkier pachymeningeal disease, and did not have associated T2 hyperintensities. Though our patient had mild improvement in pachymeningeal enhancement and thickening on MRI and metabolic activity on FDG-PET with intrathecal rituximab, she had less robust response than the other two cases. This is likely because of the greater fibrous changes that accumulated in her pachymeninges. In principally fibrotic manifestations of IgG4RD, such as thyroiditis or pachymeningitis, delayed treatment may not be able to reverse fibrotic bulk despite adequate inflammatory control due to the accumulation of non-inflammatory fibrosis over time (5, 11). She had improvement, but not normalization, in Q_alb_ and IgG index with intrathecal rituximab, likely also due to persistent pachymeningeal fibrous changes leading to impaired CSF flow. As in the previous reports, intrathecal rituximab was well-tolerated in our patient.

In conclusion, our report provides a longitudinal description of IgG4RD-HP disease progression in a patient who became refractory to intravenous rituximab and demonstrated clinical, immunologic, and radiographic improvement with intrathecal rituximab. Intrathecal rituximab should be considered as a treatment option in cases of refractory IgG4RD-HP.

## Data availability statement

The original contributions presented in the study are included in the article/Supplementary material, further inquiries can be directed to the corresponding author.

## Ethics statement

Ethical review and approval was not required for the study on human participants in accordance with the local legislation and institutional requirements. The patients/participants provided their written informed consent to participate in this study. Written informed consent was obtained from the individual(s) for the publication of any potentially identifiable images or data included in this article.

## Author contributions

DB: acquired, analyzed, and interpreted data, drafted and critically revised the manuscript, and designed figures. SH and BC: acquired data and critically revised the manuscript. BP: acquired and interpreted MRI and PET images. AO'S: acquired and interpreted PET images. AV and PJ: critically revised the manuscript. JS and NV: conceived the study, acquired, analyzed, and interpreted data, and critically revised the manuscript. All authors approved the final manuscript.

## Supplementary material

Supplementary Video 1 Video of the patient's gait after receiving several cycles of intrathecal rituximab. Prior to intrathecal rituximab therapy, the patient required a cane for ambulation. After intrathecal rituximab, she can walk independently.

## References

[1] AbdelRazek MA Venna N Stone JH. IgG4-related disease of the central and peripheral nervous systems. Lancet Neurol. (2018) 17:183–92. 10.1016/S1474-4422(17)30471-4 29413316

[2] Soares C Martins DF Pestana-Silva R Honavar M Faria O Abreu P. Intrathecal rituximab in immunoglobulin G4-hypertrophic pachymeningitis. J Neuroimmunol. (2019) 334:576997. 10.1016/j.jneuroim.2019.576997 31254930

[3] Della-Torre E Campochiaro C Cassione EB Albano L Gerevini S Bianchi-Marzoli S. Intrathecal rituximab for IgG4-related hypertrophic pachymeningitis. J Neurol Neurosurg Psychiatry. (2018) 89:441–4. 10.1136/jnnp-2017-316519 28819060

[4] Della-Torre E Galli L Franciotta D Bozzolo EP Briani C Furlan R. Diagnostic value of IgG4 Indices in IgG4-related hypertrophic pachymeningitis. J Neuroimmunol. (2014) 266:82–6. 10.1016/j.jneuroim.2013.10.008 24289956

[5] Zhang W Stone JH. Management of IgG4-related disease. The Lancet Rheumatology. (2019) 1:e55–65. 10.1016/S2665-9913(19)30017-7 38229361

[6] Khosroshahi A Wallace ZS Crowe JL Akamizu T Azumi A Carruthers MN. International consensus guidance statement on the management and treatment of IgG4-related disease. Arthritis Rheumatol. (2015) 67:1688–99. 10.1002/art.39132 25809420

[7] Carruthers MN Topazian MD Khosroshahi A Witzig TE Wallace ZS Hart PA. Rituximab for IgG4-related disease: a prospective, open-label trial. Ann Rheum Dis. (2015) 74:1171–7. 10.1136/annrheumdis-2014-206605 25667206

[8] Levraut M Cohen M Bresch S Giordana C Burel-Vandenbos F Mondot L. Immunoglobulin G4-related hypertrophic pachymeningitis: A case-oriented review. Neurol Neuroimmunol Neuroinflamm. (2019) 6:e568. 10.1212/NXI.0000000000000568 31355304 PMC6624094

[9] Reiber H Peter JB. Cerebrospinal fluid analysis: disease-related data patterns and evaluation programs. J Neurol Sci. (2001) 184:101–22. 10.1016/S0022-510X(00)00501-3 11239944

[10] Rubenstein JL Combs D Rosenberg J Levy A McDermott M Damon L. Rituximab therapy for CNS lymphomas: targeting the leptomeningeal compartment. Blood. (2003) 101:466–8. 10.1182/blood-2002-06-1636 12393404

[11] Perugino CA Stone JH. IgG4-related disease: an update on pathophysiology and implications for clinical care. Nat Rev Rheumatol. (2020) 16:702–14. 10.1038/s41584-020-0500-7 32939060

